# Preparation, Characterization, and Antioxidant Activity of Nanoemulsions Incorporating Lemon Essential Oil

**DOI:** 10.3390/antiox11040650

**Published:** 2022-03-28

**Authors:** Ting Liu, Zhipeng Gao, Weiming Zhong, Fuhua Fu, Gaoyang Li, Jiajing Guo, Yang Shan

**Affiliations:** 1 Longping Branch, Graduate School of Hunan University, Changsha 410125, China; ltchangsha98@163.com (T.L.); fhfu686@163.com (F.F.); lgy7102@163.com (G.L.); 2 International Joint Lab on Fruits & Vegetables Processing, Quality and Safety, Hunan Key Lab of Fruits & Vegetables Storage, Processing, Quality and Safety, Hunan Agriculture Product Processing Institute, Hunan Academy of Agricultural Sciences, Changsha 410125, China; 3 College of Animal Science and Technology, Hunan Agricultural University, Changsha 410128, China; gaozhipeng627@163.com (Z.G.); zhongweiming2021@163.com (W.Z.)

**Keywords:** lemon essential oil, nanoemulsions, ultrasonication, response surface methodology, antioxidant activities

## Abstract

Lemon essential oil (LEO) is a kind of citrus essential oil with antioxidant, anti-inflammatory, and antimicrobial activities, but low water solubility and biological instability hinder its industrial application. In this study, LEO was nanoemulsified to solve these problems. The preparation procedure of lemon essential oil nanoemulsions (LEO-NEs) was optimized, and the physicochemical characterization and antioxidant activities were explored. Single-factor experiments (SFEs) and response surface methodology (RSM) were conducted for the effects on the mean droplet size of LEO-NEs. Five factors of SFE which may influence the droplet size were identified: HLB value, concentration of essential oil, concentration of surfactant, ultrasonic power, and ultrasonic time. On the basis of the SFE, the RSM approach was used to optimize the preparation procedure to obtain LEO-NEs with the smallest droplet size. LEO-NEs exhibited good antioxidant activity when the HLB value was 13, content of surfactant was 0.157 g/mL, ultrasonic time was 23.50 min, and ultrasonic power was 761.65 W. In conclusion, these results can provide a good theoretical basis for the industrial application of lemon essential oil.

## 1. Introduction

Essential oils (EOs), as volatile products of secondary plant metabolism, are well known for their antioxidant [1,2], anti-inflammatory [3], and antimicrobial [4] activities. Citrus EOs have high yield and demand in EO, which are major by-products of citrus processing. Lemon essential oil (LEO) is a kind of citrus EOs, which is commonly used for flavoring and fragrance. The FDA has also deemed LEO safe for use as a preservative or flavoring agent [5]. Furthermore, some researchers reported that LEO had antioxidant activity using DPPH, ABTs, and β-Carotene bleaching assays [6,7]. The antioxidant activity of the LEO is related to the preservation of food and the prevention of diseases. Thus, it has the prospect to replace synthetic preservatives [8].

However, the greatest impediment to the widespread use of LEO is its insolubility in water, and other disadvantages include volatility, low stability, and sensitivity to the environment. LEO could be encapsulated in emulsions to reduce its hydrophobicity, but conventional emulsions are thermodynamically unstable and the components tend to separate from each other [9]. These problems can be solved by nanoemulsions (NEs) prepared using emerging nanotechnology [10]. A NE is a type of drug delivery system with a simple preparation process and stable formulation quality. It has a certain kinetic and thermodynamic stability [10], which can effectively improve the stability of the drug after emulsification on the one hand, and reduce the irritation of drug delivery on the other [11]. The droplet size of the NE is relatively small (20–200 nm) [12,13]. Meanwhile, the particle size of NEs determines its surface and interface properties. NEs with small particle size have a low particle weight and high surface-to-volume ratio, and the Brownian motion of small particle NEs can overcome gravity, which can reduce the occurrence of coalescence, aggregation, and flocculation [14,15]. However, the small droplets in oil-in-water nanoemulsions are mainly composed of oil and dispersed in water with surfactant, whereby their minimum particle size is limited by the oil [16]. Currently, the methods for preparing NEs are classified into high-energy emulsification and low-energy emulsification methods according to the physicochemical mechanism of droplet rupture. Ultrasound is a widely used high-energy process to prepare NEs. It consumes less surfactant with smaller particles compared to the low-energy method [17]. Meanwhile, it provides better control of the system and has a lower production cost than other high-energy methods (microfluidization, high-pressure homogenization) [18].

Thus, the main purpose of this study was to employ the ultrasonic method for preparing LEO-NEs with small particle size, good stability, and high antioxidant activity. SFEs and RSM were employed to prepare optimized LEO-NEs and investigate the individual effects of the independent variables on the droplet size. The findings can provide a basis for formulating and rationalizing the application of LEO-NEs and lay the foundation for their scale-up production in the cosmetics and the food industries.

## 2. Materials and Methods

### 2.1. Materials and Chemicals

Lemon was obtained from Sichuan Province. Tween-80 and Span-80 were purchased from Sinopharm Chemical Reagent Co., Ltd. (Shanghai, China). the total antioxidant capacity assay kits with DPPH and ABTS were purchased from Suzhou Comin Biotechnology Co., Ltd. (Suzhou, China). Ultrapure water (MILLI Q) was used in the experiments.

### 2.2. Methods

#### 2.2.1. Extraction and GC-MS Analysis of Lemon Essential Oil (LEO)

The method of extracting essential oil referred to Guo et al. [4]. LEO was extracted from a mixture of lemon peel and water by steam distillation. Sodium chloride was added in the extraction process, and anhydrous sodium sulfate was added to dry the essential oil after extraction. The determination of LEO components was determined according to procedures reported earlier [19]. LEO was analyzed by GC–MS using an Agilent 7890A GC with a Gerstel MPS autosampler and an Agilent 5975C MSD detector. The carrier gas was helium with a flow rate of 1 mL/min. The temperature was programmed as follows: the initial temperature of 40 °C was maintained for 1 min; the temperature was increased to 220 °C at a rate of 3 °C/min for 25 min; the final temperature of 250 °C was reached at a rate of 5 °C/min for 10 min. MS conditions were 70 eV EI and an ion source temperature of 230 °C. The mass-to-charge (*m*/*z*) range was set to 35–350 atomic units. The National Institute of Standards and Technology (NIST 08) was used to compare the data of the LEO components.

#### 2.2.2. Preparation of Lemon Oil-Based Nanoemulsions (LEO-NEs)

The LEO-NEs were formed from LEO, a mixture of two surfactants (Tween-80 and Span-80), and deionized water. A procedure for the oil–water mixtures was followed to obtain 20 mL; the pre-emulsion was centrifuged for 5 min at 10,000 rpm using a high-speed homogenizer (F6/10, Jingxin, Shanghai, China). The homogenate was processed further by an ultrasonicator (JY92-11D, Jingxin, Shanghai, China). During the ultrasonication process, samples were put in ice water for a low-temperature environment.

### 2.3. Optimization and Statistical Design of LEO-NEs

#### 2.3.1. Single-Factor Experiments (SFE)

Single-factor experiments were designed to investigate the effects of hydrophilic–lipophilic balance (HLB) value, content of Span-80 and Tween-80 (STmix), concentration of essential oil, ultrasonic time, and ultrasonic power on the mean droplet size, which can also provide a reasonable data range for the design of the response surface methodology. Specific parameters are presented in Table 1. The HLB value represented the combined affinity of hydrophilic and oleophilic groups in emulsifier molecules for oil or water [20]. Different HLB values of surfactants can contribute to the formation of two types of emulsions: water-in-oil (W/O) emulsion and oil-in-water (O/W) emulsion. To prepare the O/W LEO-NEs with hydrophilicity, an oil-in-water emulsifier with a high HLB value (8–15) was chosen. According to Nirmal et al. [21], different combinations of STmix were used to create surfactant HLB values ranging from 8–15, as shown in Table 2.

#### 2.3.2. Response Surface Methodology (RSM) Design

The levels of the independent variables to be used in the Box–Behnken designs were determined by the results of the SFE. The RSM explored the effects of the selection factor over 29 runs. In this work, the BBD with four variables (factor A was the HLB value, factor B was the STmix content, factor C was the ultrasonic time, and factor D was the ultrasonic power) at three levels (−1, 0, 1) was carried out to evaluate the effect on the dependent variable. The mean droplet size (Y) was the response value. The optimum formulation was chosen by the analysis of the RSM. The specific parameters are shown in Table 1.

### 2.4. Characterization of LEO-NEs

#### 2.4.1. Mean Droplet Size and Polydispersity Index (PDI) of LEO-NEs

The mean droplet size, particle size distribution, and PDI were measured using an NS-90 nano-granularity analyzer (Malvern Instruments Ltd., Malvern, UK). The average diameter of the particles indicated the average particle size. The intensity of particles of different diameters indicated the particle size distribution. To avoid bubbles and multiple light scattering, the LEO-NE was diluted 50-fold with ultrapure water. Three sets of measurements were performed in each sample to determine the mean droplet size and PDI of LEO-NEs in 1 mL of the diluted samples.

#### 2.4.2. Transmission Electron Microscopy (TEM) Images of LEO-NEs

The particle morphology of the LEO-NEs with a 20-fold dilution was observed by TEM (Hitachi HT-7700, Tokyo, Japan). Dilution was undertaken to prevent inter-particle aggregation.

#### 2.4.3. DPPH Radical-Scavenging Activity

The DPPH scavenging assay using 0.5 g/mL of LEO and LEO-NEs (stored for 7 days) was measured following the kit instructions. The EOs were diluted with extraction buffer in the kit. Firstly, 380 μL of Reagent 1 was added to 20 μL of sample and then shaken vigorously for 20 min. The change in absorbance was measured at 515 nm by the microplate reader (Thermo Scientific, Waltham, MA, USA). The percentage inhibition free radical scavenging rate of DPPH was calculated as follows:

DPPH scavenging activity (Inhibition%) = [(A_control_ − A_sample_)/A_control_] × 100 (1)

#### 2.4.4. ABTs Radical-Scavenging Activity

The ABTs scavenging assay of 0.5 g/mL of LEO and LEO-NEs (stored for 7 days) was performed following the kit instructions. The change in absorbance was measured at 734 nm by the microplate reader. The percentage inhibition of ABTs was calculated with the following formula:

ABTs scavenging activity (Inhibition%) = [(A_control_ − A_sample_ + A_blank_)/A_control_] × 100 (2)

### 2.5. Data Analysis

The results of the single-factor experiments were analyzed by Graphpad Prism version 8 software. The statistical analysis of the results of the response surface test was performed by Design-Expert version 13 software. All of the components, as well as their probable interactions, were examined using statistical parameters for analyses of variance (ANOVAs), such as degrees of freedom, F-ratios, and *p*-values. The model with a good fit to the data was selected (*p* < 0.05).

## 3. Results

### 3.1. Chemical Composition of the Lemon Essential Oil

A lemon-like odor liquid oil isolated by steam distillation from lemon peels was transparent and colorless. The components of the LEO identified are given in Table 3. Analysis of the volatile constituents of the LEO compounds by GC–MS identified 15 compounds that accounted for more than 0.5%, with a total of 96.36%. The major components detected in LEO were limonene (48.54%), α-pinene (30.90%), β-citral (3.65%), and β-myrcene (3.01%). As seen, the main constituents of the EO in this study were composed of monoterpene hydrocarbons (83.53%), including limonene, α-pinene, β-myrcene, and terpinolene.

### 3.2. Single-Factor Experiments

The effects of parameters on the mean droplet size of LEO-NEs were investigated using single-factor experiments, including the HLB value (8, 9, 10, 11, 12, 13, 14 and 15), concentration of essential oil (0.05, 0.06, 0.07, 0.08, 0.09 and 1 g/mL), concentration of surfactant (0.0125, 0.025, 0.05, 0.1 and 0.2 g/mL), ultrasonic power (100, 300, 500, 700 and 900 W) and ultrasonic time (0, 10, 20, 30 and 40 min). The ranges for parameter values of RSM were set to the right and left of the optimum values.

#### 3.2.1. Effect of HLB Value on the Mean Droplet Size of LEO-NEs

**Figure 1:**
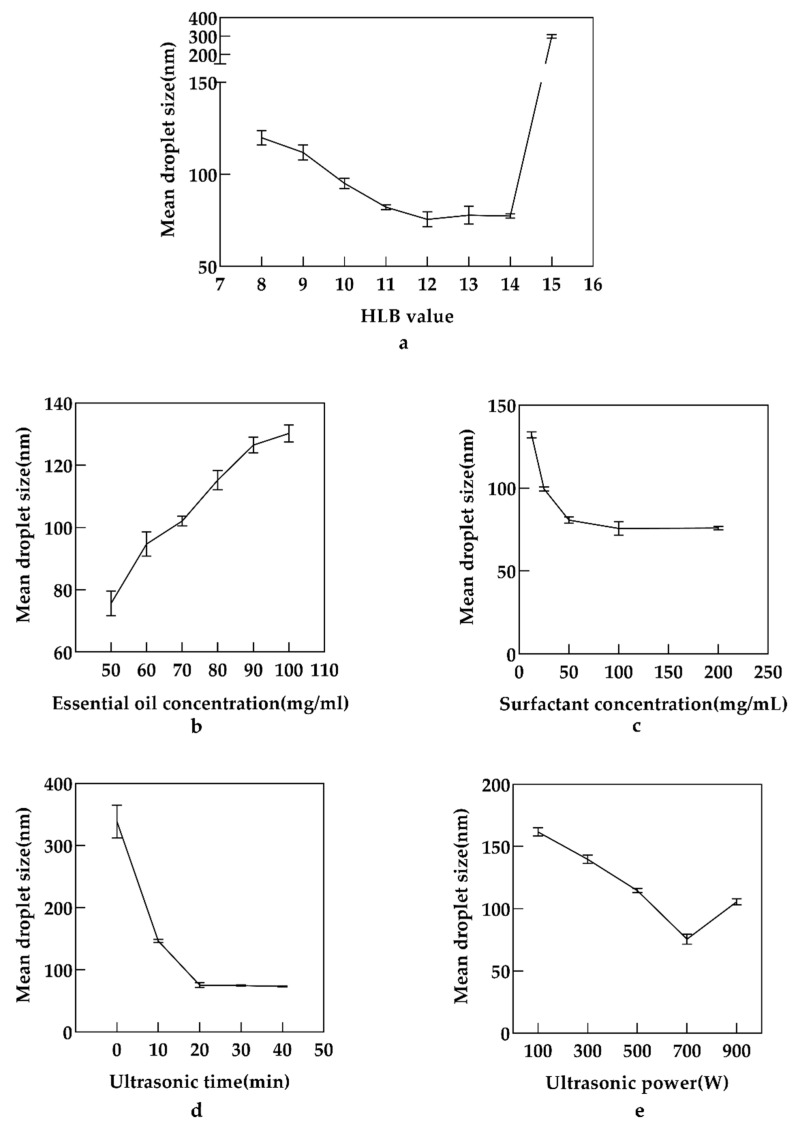
Effects of HLB value (**a**), essential oil concentration (**b**), Surfactant concentration (**c**), ultrasound time (**d**) and ultrasonic power (**e**) on the mean droplet size of NEO-NEs.

The HLB value of the surfactant can assist in identifying the best-suited stabilizer. When the HLB value of the STmix couple matches the HLB value required for the EO to form nanoemulsions, NEs with a small droplet size can be produced [22]. It was a crucial step to select an appropriate HLB value to obtain LEO-NEs with the smallest particle size. In the present work, the impact of the HLB value on the mean droplet size of LEO-NEs was studied first. As indicated in Figure 1a, when the HLB value changed from 8–12, the mean droplet size progressively declined, while the mean droplet size exhibited an upward trend when the HLB value was above 12. Furthermore, the particle size of LEO-NEs grew considerably when the HLB value increased from 14 to 15. Therefore, the optimum HLB value for the smallest droplet of LEO-NEs was 12.

#### 3.2.2. Effect of Essential Oil Concentration on the Mean Droplet Size of LEO-NEs

To explore the effect of essential oil concentration on the mean droplet size of LEO-NEs, the formulation was performed with different LEO concentrations (ranging from 0.05 to 0.1 g/mL). As shown in Figure 1b, a significant increase in the mean droplet size was observed when the LEO content was changed from 0.05 g/mL to 0.1 g/mL. The nanoemulsion with a low concentration of LEO was more suitable for production applications. According to our results, the essential oil concentration of 0.05 g/mL in LEO-NEs was finally chosen for the subsequent experiments.

#### 3.2.3. Effect of Surfactant Concentration on the Mean Droplet Size of LEO-NEs

STmix with different concentrations was used in the NEs system. As shown in Figure 1c, a sharp decrease in the mean droplet size from 133.71 to 75.66 nm was observed when STmix concentration increased from 0.0125 to 0.1 g/mL. On the other hand, it remained almost constant when increasing the surfactant concentration from 0.1 to 0.2 g/mL. Therefore, the 0.1 g/mL surfactant concentration was selected for subsequent experiments.

#### 3.2.4. Effect of Ultrasonic Time on the Mean Droplet Size of LEO-NEs

Various ultrasonic time was used to prepare the NEs, with the aim of investigating the effects on the mean droplet size of LEO-NEs. As shown in Figure 1d, when the ultrasonic time was 0, which means that the emulsion was not treated by ultrasound, the mean droplet size fluctuated over a wide range, and the repeatability of the experiment was poor. Meanwhile, a layering phenomenon was observed after staying still at room temperature overnight. The smallest particle size was observed at the ultrasonic time of 20 min. The increase in ultrasonic time can promote the integration of water and oil. Longer ultrasonic times, on the other hand, may result in higher degradation or disintegration of bioactive chemicals in LEO, as well as energy waste [23]. Therefore, ultrasonic time of 20 min was selected for the subsequent studies considering both saving energy and achieving the best results.

#### 3.2.5. Effect of Ultrasonic Power on the Mean Droplet Size of LEO-NEs

To study the effects of ultrasonic power on the mean droplet size of LEO-NEs, the preparation process was carried out with different ultrasonic powers ranging from 100 to 900 W. As shown in Figure 1e, the value of droplet size decreased with the increase in ultrasonic power from 100 to 700 W. However, the particle size increased instead when the ultrasonic power was increased from 700 to 900 W. Excessive ultrasonic power may induce a rise in the number of bubbles in solvents during cavitation, lowering the efficiency of the ultrasound energy delivered into the medium [24]. As a result, the ultrasonic power of 700 W was chosen for further experiments.

### 3.3. Response Surface Optimization of LEO-NEs

The preparation process was further optimized using BBD experiments to obtain the best experimental parameters. The BBD of the response surface was used to optimize the formulation and preparation of LEO-NEs. The following regression equation model was obtained by regression analysis:

Y = 88.50 − 14.85 A + 6.87 B − 31.66 C − 10.15 D − 5.40 AB + 4.88 AC + 2.60 AD − 17.62 BC + 6.21 BD + 3.01 CD + 3.93 A^2^ + 19.65 B^2^ + 14.46 C^2^ + 3.34 D^2^ − 26.38 A^2^B + 10.80 A^2^C − 0.1079 A^2^D + 13.10 AB^2^ + 13.07 AC^2^ + 2.67 B^2^C − 11.58 B^2^D + 2.48 BC^2^ (3)

As shown in Table 4, the generation of a model with no significant lack of fit implied it suitability. The *R^2^* > 90% indicated that the model could accurately reflect the change in the response value when the fitness was high. The coefficient of variance (CV), which is the ratio of the estimated standard error to the mean of the observed responses, is related to the model reproducibility. Our model had a CV of 9.97% (<10%), which is usually considered to be sufficiently reproducible. The ratio of adequate precision reflects the ratio of response to the deviation, and its value was 11.840 (>4), indicating an adequate signal. 

An analysis of variance of the regression coefficient revealed that C was extremely significantly different (*p* < 0.01), while A significantly differed in its linear effect (*p* < 0.05), remaining factors indicated a non-significant difference. The main effect relationship of each factor could be ranked as ultrasonic time > HLB value > ultrasonic power > surfactant content. Among interaction effects, B^2^ had extremely significant differences (*p* < 0.01), while BC and C^2^ significantly differed (*p* < 0.05). The remaining effects were not significant (*p* > 0.1). The experimental values of mean droplet size of nanoemulsions are presented in Table 5.

**Figure 2:**
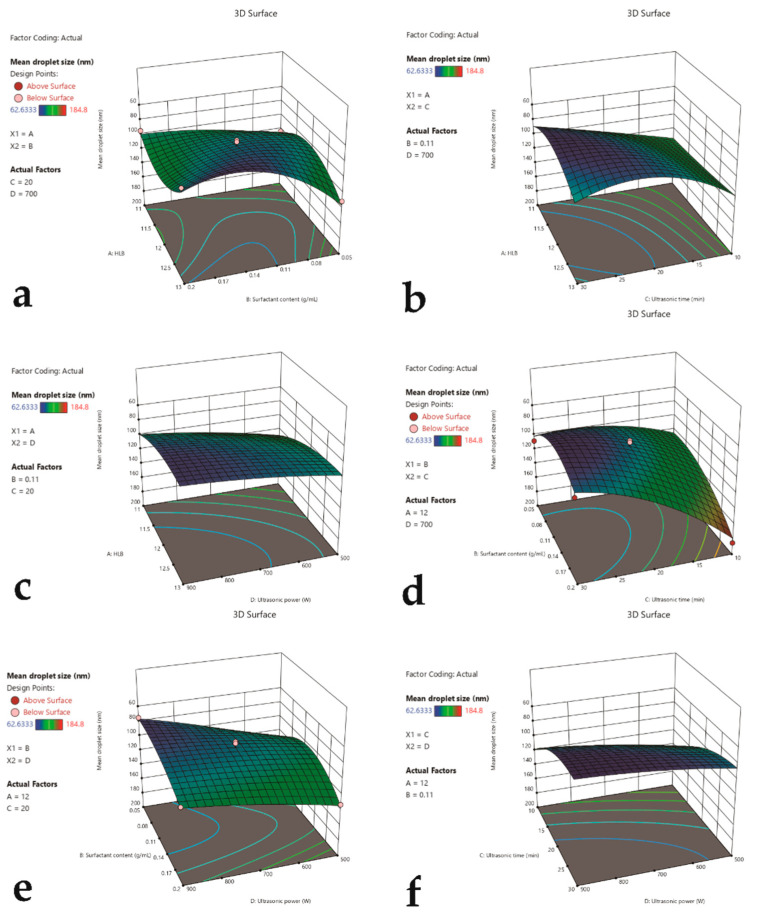
Response surface plot showing the significant (*p* < 0.05) interaction effect for mean droplet size as a function of (**a**) HLB value of STmix and STmix content, (**b**) HLB value of STmix and ultrasonic time, (**c**) HLB value of STmix and ultrasonic power, (**d**) STmix content and ultrasonic time, (**e**) STmix content and ultrasonic power, and (**f**) ultrasonic time and ultrasonic power.

Finally, the response surface was plotted using Design-expert 13. The effect of the two-factor interaction on the size of the mean droplet is intuitively shown in Figure 2. The response surface slope was steeper in Figure 2d, indicating that the interplay of surfactant content and ultrasound time had a bigger impact on LEO-NE particle size. The gradient of the response surface was moderate, as shown in Figure 2c, showing that the interaction of HLB value and ultrasonic power had less of an effect on the droplet size.

### 3.4. Physicochemical Properties and Stability of LEO-NEs

**Figure 3:**
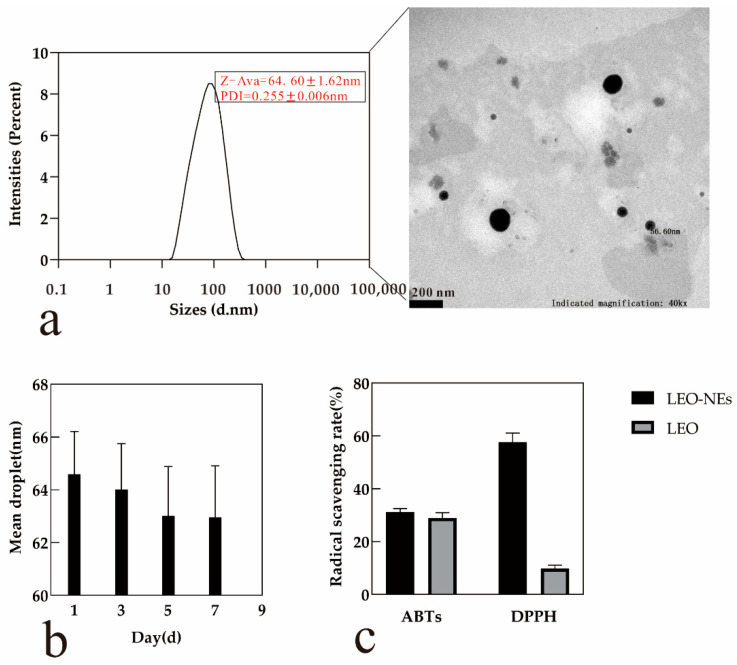
Physicochemical Properties and Stability of LEO-NEs: (**a**) particle size distribution and transmission electron microscopy (TEM) of droplets of LEO-NEs; (**b**) effect of storage time on particle size of LEO-NEs; (**c**) antioxidant activity comparison between LEO-NEs and LEO.

The above regression model was used to generate the optimum process parameters and validation results. When the HLB value (A) was 13, surfactant content (B) was 0.157 g/mL, ultrasonic time (C) was 23.50 min, and ultrasonic power (D) was 761.65 W, the predicted minimum mean droplet size was 66.82 nm. The actual mean particle diameter was 64.60 nm, and the PDI was 0.255. The particle size distribution of NEO-NEs is shown in Figure 3a. Then, the morphological changes and the changes in particle size of NEs during the storage period were observed, and the difference in antioxidant activity between emulsion and essential oil was compared.

#### 3.4.1. Morphological Observation of LEO-NEs

TEM was used to describe the morphology of the LEO-NEs, as shown in Figure 3a, the droplets were well distributed and spherical. However, the diameters of the particles in the NEs were not the same, ranging from 50–100 nm, in line with those reported by the particle size meter.

#### 3.4.2. Changes in Particle Size of LEO-NEs during the Storage Period

Figure 3b demonstrates the changes in the mean droplet of LEO-NEs for 1 week at a storage temperature of 25 °C. The particle size of the LEO-NEs changed little during a week, ranging from 62.96 nm to 64.60 nm.

#### 3.4.3. Antioxidant Activity of LEO-NEs

The DPPH and ABTs assays were used to measure the free-radical-scavenging potential to compare the difference between LEO-NEs and EO at the same concentration (0.05 g/mL). It has been shown that EOs exhibit antioxidant activities due to large amounts of polyphenol compounds. Figure 3c shows that the antioxidant activity of the LEO-NEs was higher than that of LEO with the same concentration, whereby the inhibition of DPPH free radicals by LEO-NEs (57.61%) was much better than that by LEO (8.74%), but the inhibition of ABTs free radicals by LEO-NEs (31.74%) was similar to that by LEO (30.61%).

## 4. Discussion

Coinciding with the reports by other Hirai et al. [25], Aguilar et al. [26], Perdones et al. [27] and Campolo et al. [28], limonene was the most abundant component in LEO while its content may vary. LEO was rich in constituents with monoterpene structure (limonene, α-pinene, etc.) which have been proven to possess antioxidant activity [29]. For example, limonene was shown to prevent neuronal suffering [30], oxidative stress on lymphocytes, and mitochondrial dysfunction [29] through its antioxidant activity. In addition, LEO components present in other studies were not detected in this experiment such as β-phellandrene [25], camphene, and sabinene [26]. Differences in LEO composition may be due to differences in geographic location, environmental factors, plant age, developmental stage, harvest time, extraction site, and extraction method [31].

In this study, LEO-NEs were prepared by the ultrasonic method using STmix as an emulsifier. The influence of each factor was studied by SFEs. Firstly, when the HLB values of STmix ranged from 8–14, the mean droplet size of NEs was less than 200 nm, while NEs could not be formed when HLB was 15. The large range of suitable HLB values means that many kinds of surfactants can be used to prepare LEO-NEs. Tween-80 was not suitable for this experiment; however, it was also used to make lemon LEO-NEs in other experiments. Mossa et al. [13] reported that the droplet size of LEO-NEs was 131.9 nm, while the particle size of the LEO-NEs was 181.5 nm in the study of Yazgan [32]. Although these studies were able to form NEs with Tween-80, the particle sizes were larger than 100 nm. Furthermore, the droplet diameter of LEO-NEs was 91 nm [33] and 135 nm [34] when produced with Tween-80 using the high-pressure homogenizer method. These results indicate that different essential oil components and different emulsification methods may lead to different particle sizes when constructing NEs.

When the concentration of essential oil was 0.05–0.1 g/mL, the particle size increases with the increase in concentration of essential oil, indicating its greater impact on particle size. However, previous studies showed that the particle size would not increase when the essential oil exceeds a certain amount if the concentration of surfactant micelles remains not changed, because of a saturation with lemon oil, whereby any further lemon oil droplets added to the nanoemulsions would not dissolve [35]. This phenomenon did not occur in our experiments because the concentration range of LEO was not large enough. An increase in surfactant concentration can also lead to a decrease in particle size. The surfactants can affect inter-particle interactions in emulsions, whereby a the higher surfactant concentration results in weaker inter-particle interactions and smaller droplets formed [36]. The effect of surfactant concentration on mean particle size may be related to the surfactant dose required to cover the surface of the formed droplets, whereby self-emulsification would be more dependent on surfactant concentration [37]. In the process of ultrasonic preparation of NEs, the particle size did not decrease when the surfactant concentration increased to a certain amount. In addition, the dependence of the mean particle size on surfactant concentration did not depend strongly on storage time and temperature [12].

Ultrasonic cavitation is a feasible and energy-efficient method for preparing NEs, which offers improvements in terms of stability and decreases the Ostwald ripening rate. During the ultrasonication processes, soundwave energy causes cavities and sinusoidal pressure variations in the liquid–liquid interphase, resulting in a shockwave action on the particle surface and a reduction in particle size [38]. The particle size of nanoemulsions prepared with the ultrasonic method is generally determined by the sonication time and sonication power, but is insensitive to ultrasonication amplitude [39]. Understanding the dynamic routes is critical for reducing processing time and avoiding energy oversupply. When the ultrasonic time reached a certain value, the particle size reached the minimum. Increasing the ultrasonic time would not lead to a significant change in particle size. The increase in ultrasonic power led to a decrease and then increase in particle size, coinciding with the report of Kentish et al. [40]. In addition, Floris et al. [41] reported that high ultrasonic power may destroy bioactive substances.

The small size of the particles in NEs would result in less agglomeration or precipitation and higher stability of the system [42,43]. RSM was used to optimize NEs to obtain the smallest droplet size. The interaction between surfactant concentration and ultrasonic time had the greatest effect on particle size. However, the particle size did not decrease indefinitely, as it was limited by the ingredients of the essential oil. The optimal preparation conditions obtained by RSM were similar to those obtained by SFE, and the conditions predicted by RSM were relatively more precise.

Due to the mass transfer of oil molecules, droplets in NEs change from smaller droplets to larger droplets through an intermediate water phase, which is called Ostwald ripening. Ostwald ripening leads to droplet growth and phase separation [44]. From the TEM image and the particle size change during storage, particle diameter does not exceed 200 nm; hence, the ripening phenomenon was not serious in LEO-NEs. However, the TEM images revealed that the diameters of the particles in the nanoemulsion were not the same. The TEM image was similar to that presented by Kaur et al. [45] and Zhong et al. [46]. In previous studies, the structure of NE was presented a spherical substance consisting of several small spherical packets [46]. The particle size of LEO-NEs had the tendency to decrease in 1 week, possibly due to the EOs in the NEs undergoing a small amount of evaporation, thereby reducing the content of essential oil. In the study of Zhong et al. [46], there was a tendency for the particle size to increase with storage time, which may have been due to Ostwald ripening.

The prepared NE was not only stable but also had sustained-release activities. The study of antioxidant activities is essential as reflected in the reduction in reactive oxygen species (ROS) in the food and cosmetics industries. We could find that the encapsulation of essential oils in NEs helped to enhance their antioxidant activities when comparing the antioxidant activity of essential oils and NEs. DPPH scavenging activity refers to the ability to reduce the stable DPPH free radical to its reduced form DPPH-H [47]. ABTs scavenging activity refers to the ability to decolorize the radical cation (ABTS^•+^) [48]. Due to the different principles of determination, the two results are not necessarily related. The different methods employed to indicate antioxidant activity can comprehensively profile the antioxidant activities of LEO-NEs. According to a previous study [6], the DPPH radical scavenging activity and ABTs radical-scavenging activity of pure LEO were 32.85% and 41.57% respectively. These results are close to the antioxidant capacity of LEO-NEs in our study, but the composition and determination method of the essential oil had an impact on the results. In addition, the antioxidant activity of LEO and LEO-NEs may be due to components in LEO with antioxidant activity. However, The antioxidant activity of LEO should not only consider the primary constituents [49]. The main antioxidant components in lemon essential oil need to be further studied.

## 5. Conclusions

This study explored the optimum preparation procedure of LEO-NEs using SFEs and RSM. The optimal parameters were as follows: HLB value of 13, surfactant content of 0.157 g/mL, ultrasonic time of 23.50 min, and ultrasonic power of 761.65 W. The optimized mean droplet size was 64.60 nm. In addition, the TEM images and storage results demonstrated the good dispersion and stability of LEO-NEs. The antioxidant activity experiments showed that LEO-NEs had better antioxidant capacity than essential oils. Some of the characteristics of LEO-NEs investigated in this study and future endeavors may lay the foundation for the practical application of antioxidant activity and other biological activities of LEO-NEs.

## Figures and Tables

**Table 1:** Variables of single-factor experiments (SFE) and Response surface methodology (RSM).

Factors of SFE	Variables							
HLB value of STmix	8	9	10	11	12	13	14	15
concentration of LEO (g/mL)	0.05	0.06	0.07	0.08	0.09	0.1		
STmix content (g/mL)	0.0125	0.025	0.05	0.1	0.2			
ultrasonic time (min)	0	10	20	30	40			
ultrasonic power (W)	100	300	500	700	900			
**Independent Variables of RSM**	**Levels**							
	−1			0		1		
A: HLB value of STmix	11			12		13		
B: content of STmix (g/mL)	0.05			0.125		0.2		
C: ultrasonic time (min)	10			20		30		
D: ultrasonic power (W)	500			700		900		

**Table 2:** Different combinations of Span 80 and Tween 80 used to create surfactant HLB value.

HLB	Span 80 (%)	Tween 80 (%)
8	65.4	34.6
9	56.9	43.1
10	46.7	53.3
11	37.4	62.6
12	28	72
13	18.7	81.3
14	9.3	90.7
15	0	100

**Table 3:** Chemical composition (%) of the essential oil isolated from lemon peels.

No	Main Component	Content (%)	Classification
1	Limonene	48.54	Monoterpene Hydrocarbons
2	α-Pinene	30.9	Monoterpene Hydrocarbons
3	β-Citral	3.65	Monoterpene aldehydes
4	β-Myrcene	3.01	Monoterpene Hydrocarbons
5	Neryl Acetate	1.74	Oxygenated Terpenes
6	β-Bisabolene	1.31	Sesquiterpene Hydrocarbons
7	α-Terpineol	1.11	Oxygenated Terpenes
8	Terpinolene	1.08	Monoterpene Hydrocarbons
9	α-bergamotene	0.97	Sesquiterpene Hydrocarbons
10	Thujane	0.85	Monoterpene alkanes
11	Caryophyllene	0.72	Sesquiterpene Hydrocarbons
12	4-Terpineol	0.68	Oxygenated Terpenes
13	Geraniol	0.68	Oxygenated Terpenes
14	Nerol	0.61	Oxygenated Terpenes
15	Valencene	0.51	Sesquiterpene Hydrocarbons

**Table 4:** ANOVA of RSM outcome ^α^.

Source	Sum of Squares	df	Mean Square	F-Value	*p*-Value	
Model	19,794.11	22	899.73	8.11	0.0077	significant
A-HLB	881.89	1	881.89	7.95	0.0304	
B-Surfactant content	188.81	1	188.81	1.7	0.2398	
C-Ultrasonic time	4009.95	1	4009.95	36.14	0.001	
D-Ultrasonic power	411.79	1	411.79	3.71	0.1023	
AB	116.53	1	116.53	1.05	0.3449	
AC	95.39	1	95.39	0.8598	0.3896	
AD	26.95	1	26.95	0.243	0.6396	
BC	1241.27	1	1241.27	11.19	0.0155	
BD	154.36	1	154.36	1.39	0.2828	
CD	36.29	1	36.29	0.3271	0.5881	
A^2^	100.41	1	100.41	0.905	0.3782	
B^2^	2505.85	1	2505.85	22.59	0.0032	
C^2^	1357.05	1	1357.05	12.23	0.0129	
D^2^	72.53	1	72.53	0.6538	0.4496	
Residual	665.65	6	110.94			
Lack of Fit	514.75	2	257.38	6.82	0.0514	not significant

^α^*R^2^* = 0.97; adj. *R^2^* = 0.85; C.V. (%) = 9.97; adequate precision = 11.84.

**Table 5:** Experimental values of mean droplet size of nanoemulsions obtained from BBD experimental design.

Run	HLB	Surfactant Content (g/mL)	Ultrasound Time (min)	Ultrasound Power (W)	Mean Droplet Size (nm)
1	12	0.2	20	500	132.53
2	12	0.2	20	900	101.51
3	12	0.125	10	900	119.93
4	11	0.2	20	700	94.85
5	11	0.125	20	900	104.0
6	13	0.125	30	700	87.79
7	11	0.125	20	500	129.70
8	12	0.05	20	900	75.35
9	13	0.125	20	900	79.50
10	13	0.125	20	500	94.81
11	12	0.125	30	500	76.90
12	13	0.125	10	700	119.75
13	12	0.125	10	500	146.25
14	12	0.05	20	500	131.22
15	12	0.125	20	700	94.79
16	13	0.2	20	700	80.56
17	11	0.125	30	700	81.59
18	12	0.125	20	700	92.41
19	12	0.2	10	700	184.80
20	12	0.125	20	700	91.35
21	12	0.05	10	700	130.87
22	12	0.125	20	700	83.11
23	12	0.125	30	900	62.63
24	11	0.05	20	700	123.07
25	12	0.05	30	700	108.12
26	13	0.05	20	700	130.37
27	12	0.125	20	700	80.830
28	12	0.2	30	700	91.587
29	11	0.125	10	700	133.083

## Data Availability

Data is contained within the article.
